# Postoperative constrictive pericarditis caused rupture of lymphatic collaterals: a rare etiology of chylothorax

**DOI:** 10.1186/s44215-023-00092-9

**Published:** 2023-08-09

**Authors:** Yasuhito Nakamura, Kiyoshi Doi, Ryo Fujii, Hiroki Ogura, Etsuji Umeda, Takayoshi Kato, Osamu Sakai, Katsuya Shimabukuro

**Affiliations:** Department of Cardiovascular Surgery, Graduate School of Medicine, Gifu University, Gifu, Japan

**Keywords:** Constrictive pericarditis, Chylothorax, Cardiac surgery, Pericardiectomy

## Abstract

**Background:**

Chylothorax after cardiovascular surgery is primarily caused by direct injury to the thoracic duct or its branches, and occurs in early postoperative period. In the present case, we describe a rare case of chylothorax manifesting three months after surgery secondary to constrictive pericarditis.

**Case presentation:**

A 71-year-old man underwent mitral valve replacement, tricuspid valve annuloplasty, and the maze procedure. He developed acute perioperative myocardial infarction on postoperative day one due to plaque rupture in the left anterior descending artery and underwent percutaneous coronary intervention. Although the patient was discharged on postoperative day 36, he required readmission on postoperative day 83 because of right side chylothorax associated with constrictive pericarditis. Lymphangiography revealed thoracic duct interruption and development of lymphatic collateral vessels via the right hilum. Single-photon emission computed tomography revealed abnormal tracer accumulation in the right hilum, suggesting a lymphatic leakage site. A catheter study indicated biventricular dip and plateau patterns with a reduced cardiac index (1.6 L/min/m^2^) and elevated central venous pressure (18 mmHg). Conservative treatment for chylothorax, including a low-fat diet and continuous drainage with chest tube, was unsuccessful. Drainage of chyle at approximately 500 mL/day continued. On hospitalization day 50, complete pericardiectomy via median sternotomy was performed because the patient’s cardiac function deteriorated. The central venous pressure dramatically decreased, and the chylothorax gradually subsided. There was no recurrence of symptoms 1 year postoperatively.

**Conclusions:**

Chylothorax associated with constrictive pericarditis subsequent to cardiac surgery is extremely rare. Although conservative management failed, the present case was successfully treated via pericardiectomy alone and did not require any additional precedures, such as thoracic duct ligation via a right thoracotomy.

## Background

Chylothorax occurs 1–5% of cases after cardiovascular surgery [1]. The most frequent etiology of postoperative chlothorax is direct injuries to the thoracic duct or its branches, resulting in the accumulation of chylomicrons-containing lymphatic fluid in the thoracic cavity soon after surgery [2]. Iatrogenic obstruction of the thoracic duct might increase mediastinal lymphatic pressure, leading to rupture of the branches and chyle leakage [3]. However, this etiology is less common, because development of lymphatic collateralization usually decreases the pressure within 2 weeks [4]. Therefore, surgical ligation of the thoracic duct can be considered a treatment option for chylothorax [5]. Constrictive pericarditis is a condition characterized by a thickened pericardium, resulting in restricted venous return and reduced cardiac output [6], that occasionally develops into chylothorax, although the precise mechanism is not well understood. We present a rare case of chylothorax, secondary to postoperative constrictive pericarditis, manifesting 3 months after mitral valve replacement. To our knowledge, this is the first report that elucidates the precise mechanism underlying pericarditis-induced chylothorax.

## Case presentation

**Fig. 1:**
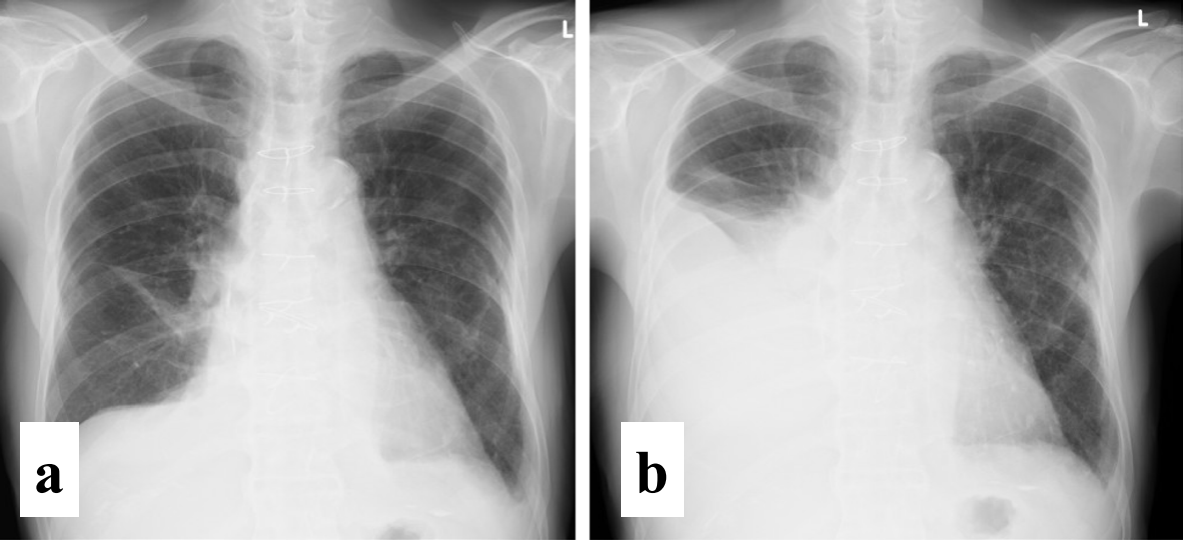
Chest radiograph. **a** There is no pleural effusion at the time of discharge (postoperative day 36). **b** A large amount of effusion is observed in the right pleural cavity at the time of readmission (postoperative day 83)

A 71-year-old man had a history of diabetes, chronic kidney disease, percutaneous coronary intervention (PCI), and persistent atrial fibrillation. Three months prior, he had undergone mitral valve replacement, tricuspid valve annuloplasty, and the maze procedure using bipolar radiofrequency ablation (Aricure® Inc., West Chester, OH, USA). Preoperative coronary angiography showed only diffuse 50% stenosis on the mid left anterior descending artery (LAD); therefore we did not perform coronary artery bypass grafting. However, the patient developed acute myocardial infarction due to plaque rupture of the LAD lesion on postoperative day 1 and underwent PCI. Aspirin, clopidogrel, and warfarin were administered in the first postoperative month, after which only clopidogrel was withdrawn. On postoperative day 13, the echocardiogram showed a moderate amount of pericardial effusion (10 mm) with prolonged elevation of inflammatory markers (white cell count, 10,400 × 10^3^/mL; C-reactive protein level, 2.66 mg/dL), indicating post-pericardiotomy syndrome; hence. administration of colchicine was initiated. Owing to absence of cardiac tamponade, pericardiocentesis was not performed. The systemic inflammatory response gradually subsided (white cell count, 7570 × 10^3^/mL; C-reactive protein level, 2.02 mg/dL), and the patient was discharged on postoperative day 36. At discharge, the echocardiogram showed a small amount of pericardial effusion (5 mm), whereas the chest radiography did not show pleural effusion (Fig. 1a).

On postoperative day 83, the patient required hospital readmission because of respiratory distress. Radiography revealed a large amount of pleural fluid on the right side (Fig. 1b). Analysis of the milky yellowish fluid confirmed the presence of chlyothorax. Catecholamine administration was initiated because of significant hypotension and peripheral edema.

**Fig. 2:**
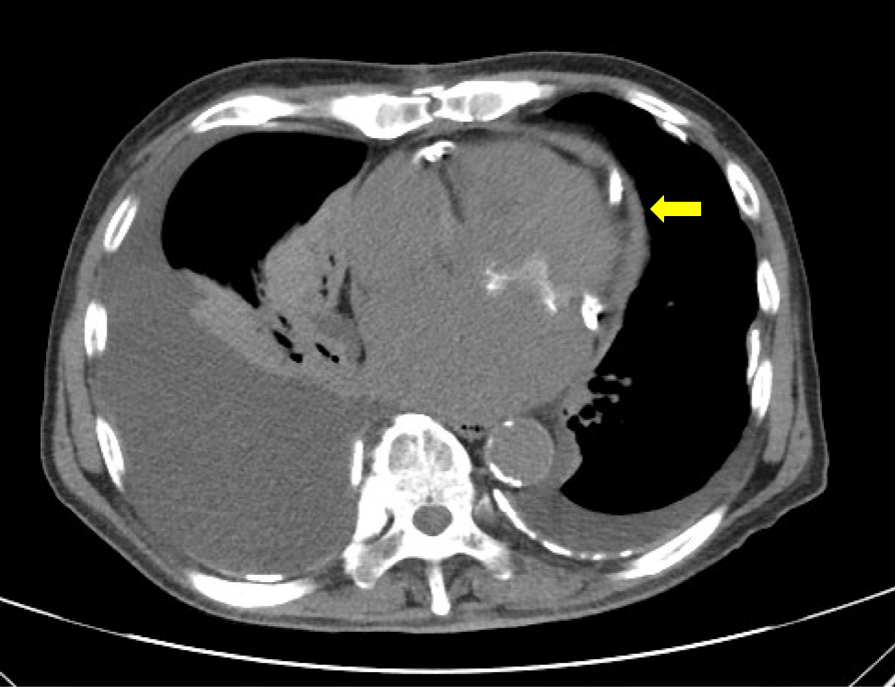
Computed tomography on readmission. There is a large amount of right pleural effusion. The pericardium is thickened and accompanied by calcification (arrow)

**Fig. 3:**
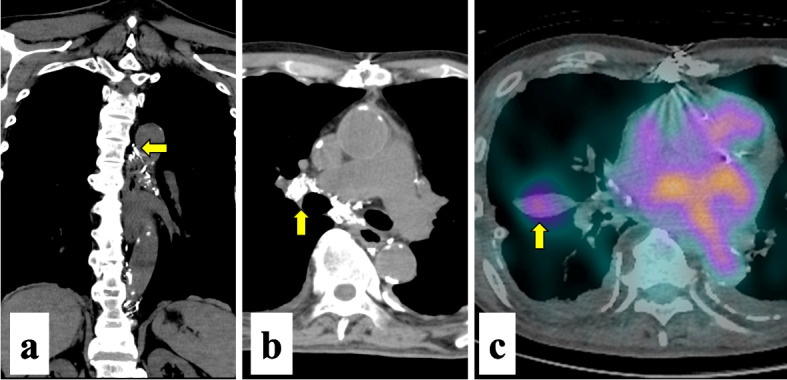
Mechanism of pericarditis-induced chylothorax. **a** Lymphangiography shows that the thoracic duct is interrupted at the Th5/6 level (arrow) by postoperative pericardial inflammation. **b** Delayed lymphangiography images showing that obstruction of the thoracic duct leads to development of lymphatic collaetarls via the tracheal bifurcation and right hilum (arrow). **c** Single-photon emission computed tomography showing abnormal tracer accumulation in the right hilum between the middle and lower lobes (arrow), suggesting a lymphatic leakage from ruptured lymphatic collaterals

Chest computed tomography revealed a large amount of right-sided pleural effusion and thickening of the pericardium with calcification (Fig. 2). Echocardiography revealed increased brightness of the inferior and posterior epicardium, paradoxical motion of the interventricular septum, and respiratory fluctuations in atrioventricular valve inflow. Bicardiac catheterization revealed biventricular dip and plateau patterns, and equal diastolic pressure in both ventricles. The cardiac index was reduced to 1.6 L/min/m^2^, and the central venous pressure was elevated to 18 mmHg. These findings led to the diagnosis of constrictive pericarditis. After admission, a chest tube was iserted in the right pleural cavity. Although the patient was on a low-fat diet, drainage of chyle at approximately 500 mL/day continued. Lymphangiography was performed on hospitalization day 22 to detect the leakage site of the chyle. The thoracic duct was interrupted at the Th5/6 level (Fig. 3a). On delayed images, lymphatic collaterals via the tracheal bifurcation and right hilum were observed (Fig. 3b). Single-photon emission computed tomography (SPECT) revealed abnormal tracer accumulation in the right hilum between the middle and lower lobes (Fig. 3c), suggesting a lymphatic leakage site.

**Fig. 4:**
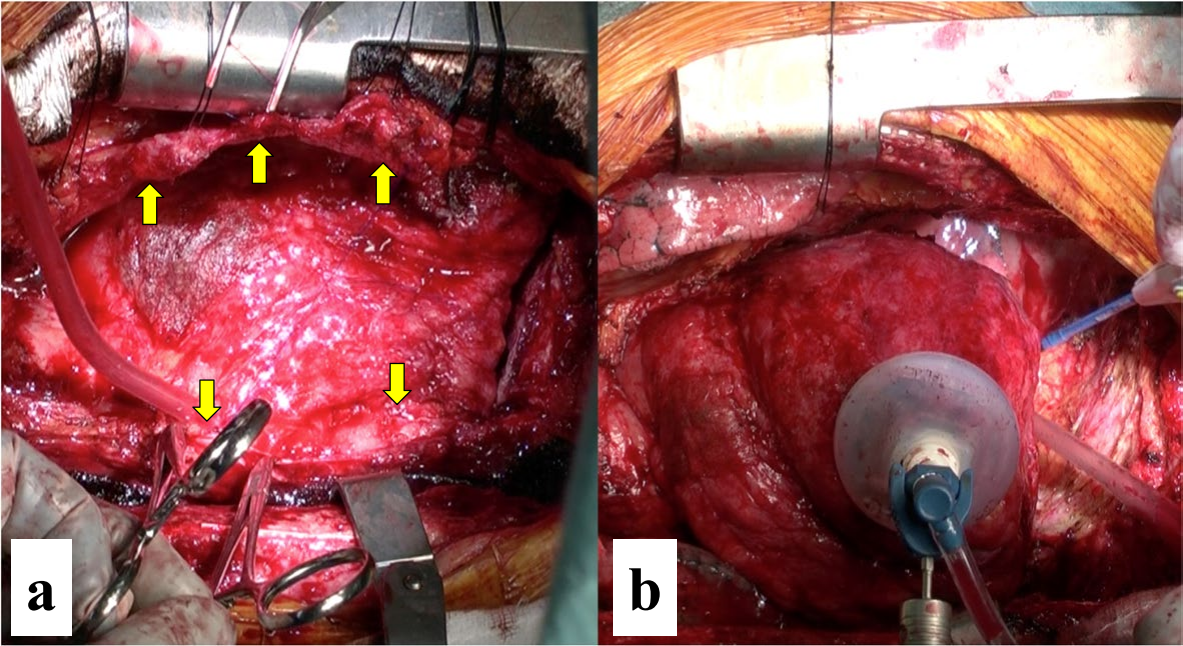
Intraoperative images. **a** The heart is encapsulated in the very thick pericardium. The pericardial flaps are excised approximately 2 cm anterior to the right and left phrenic nerves (arrows). **b** The dissection continues posteriorly until the entire left ventricle is freed. Posterior piece of pericardial flap is also excised approximately 2 cm posterior to the left phrenic nerve

As it was difficult to control heart failure and chylothorax by medical therapy alone, the patient was scheduled for surgical pericardiectomy and exploration of the chyle leakage point on hospitalization day 50. Under general anesthesia, a thoracoscope was inserted in the right seventh intercostal space, and the inside of the right thoracic cavity was inspected. Milk was administrated via the nasogastric tube prior to surgery. Nonetheless, a chyle leakage point was not identified. After median sternotomy, a complete pericardiectomy was performed without extracorporeal circulatory support (Fig. 4a, b). The cardiac index and central venous pressure improved from 1.6 L/min/m^2^ and 23 mmHg to 2.3 L/min/m^2^ and 15 mmHg, respectively. The chylothorax gradually subsided, and the right chest tube was removed 12 days after pericardiectomy. The patient was discharged from the hospital on postoperative day 32. At 1 year postoperatively, he was recurrence free.

## Discussion

Postoperative constrictive pericarditis is a rare complication that occurs in 0.2–0.3% open-heart surgery cases [7]. Direct trauma to the pericardium occasionally causes persistant local inflammation, resulting in tight adhesion and pericardial fibrosis. The timing of presentation varies between one month and several years postoperatively [8]. Its typical clinical manifestations is progressive low cardiac output syndrome due to encapsulation by thickened pericardium. Peripheral edema and pleural effusion with elevated venous pressure are often observed due to reduced venous return [6]. However, chylothorax secondary to constrictive pericarditis is very rare, and its underlying mechanism is unclear.

The risk factors leading to postoperative constrictive pericarditis are not well known. As reported by Matsuyama, anticoagulation therapy with warfarin could have increased the risk of pericarditis in the present case [9]. Because pericarditis after myocardial infarction (Dressler syndrome) rarely requires pericardiectomy [10], the major cause of the severe constriction in the present case seems to be cardiac surgery. Althogh colchicine reduces the risk of pericarditis [11], it was not effective in the present case.

For the present case, we suggest two possible mechanisms of the pericarditis-induced chylothorax. First, mechanical obstruction of the thoracic duct increased the collateral lymphatic flow and pressure, resulting in overflow chyle leakage. As shown in lymphangiography and SPECT images (Fig. 3a–c), obstruction of the thoracic duct led to development of collateral vessels via the right hilum for lymphatic drainage with abnormal tracer accumulation, suggesting a leaking site. Long standing pericardial inflammation might involve the surrounding tissue and interrupt the thoracic duct [12]. Because the maze procedure was performed in the present case, radiofrequency ablation could cause chylothorax secondary to thoracic duct obstruction as reported by Navaratnarajah [13]. Surgical reconstruction of the occluded thoracic duct could have resolved chylothorax [3]. However, thoracic duct obstruction rarely causes chylothorax because development of sufficient collateralization achieves decompression of the lymphatic system within 2 weeks [4].

Second, high central venous pressure might have limited the return of mediastinal lymphatic flow to the systemic circulation (functional obstruction) and the consequently elevated lymphatic pressure resulted in rupture of branches. This hypothesis is supported by case reports describing high central venous pressure secondary to thrombosis causing chylothorax [14].

To our knowledge, 10 cases of pericarditis-induced chlylothorax, including the present case, have been reported to date [12, 15–22]. None, except the present case, was associated with cardiac surgery. Lymphangiography was performed in five cases; of these, one case reported no thoracic duct obstruction, two cases showed mechanical obstruction, and two cases showed functional obstruction. Eight of the 10 cases were successfully treated via pericardiectomy alone; however, two required additional ligation of the thoracic duct. Therefore, the second mechanism (functional obstruction) seems to play a major role in development of pericarditis-induced chylothorax.

There are no definitive guidelines for chylothorax treatment. Conservative management is generally attempted in such cases [2]. This includes insertion of a thoracic drainage tube and complete oral intake cessation with total parenteral nutrition (TPN). However, TPN carries a risk of catheter-related bloodstream infection. Therefore, a low-fat diet strategy is currently advocated because it has demonstrated a comparable success rate to cure chylothorax [23]. Octreotide is another therapeutic option for chylothorax [24]. However, octreotide potentially compromises blood sugar control in patients with diabetes and was not applied in the present case. In addition to the diagnostic role of identifying the lymphatic leakage site, lymphangiography often reduces lymphatic leaks because lipiodol causes an inflammatory reaction that seals the extravasation from the broken lymphatic vessels [25]. However, in the present case, chyle leak did not decrease even after lymphangiography. Surgical treatment was considered when conservative management was unsuccessful [2]. As shown in the present case, identification of the leakage site during surgery is generally difficult and direct clipping of lymphatic vessels is likely to create a tear and consequently lead to a new iatrogenic chyle leak [26]. Thus, mass ligation of the thoracic duct via right thoracotomy at the level of the diaphragm is recommended [5]. Fortunately, in the present case, pericardiectomy alone dramatically decreased the chyle leakage because the reduction in central venous pressure enhanced mediastinal lymphatic drainage into the systemic circulation, further reduced the high lymphatic pressure and consequently stopped leakage from the ruptured lymphatic branch at the right hilum.

## Conclusion

Chylothorax associated with constrictive pericarditis after cardiac surgery is extremely rare. Although conservative management failed, the present case was successfully treated via pericardiectomy alone and did not require any additional procedures, such as thoracic duct ligation via a right thoracotomy.

## Data Availability

The data underlying this article will be shared on reasonable request to the corresponding author.

## Abbreviations

LAD: Left anterior descending artery
PCI: Percutaneous coronary intervention
SPECT: Single-photon emission computed tomography
TPN: Total parenteral nutrition
